# Reducing Post-Operative Hospital Length of Stay following Uncomplicated Appendectomy in Pediatric Patients: A Prospective Clinical Study

**DOI:** 10.3390/healthcare12040474

**Published:** 2024-02-14

**Authors:** Michelle A. Jeski, Jennifer D. Stanger, Melissa S. Schafer, Andrew W. Osten, Gregory P. Conners

**Affiliations:** 1 Department of Nursing, Upstate Golisano Children’s Hospital, Upstate Medical University, Syracuse, NY 13210, USA; jeskim@upstate.edu; 2 School of Nursing, Quinnipiac University, Hamden, CT 06518, USA; 3 Department of Surgery, Norton College of Medicine, Upstate Golisano Children’s Hospital, Upstate Medical University, Syracuse, NY 13210, USA; 4 Department of Pediatrics, Norton College of Medicine, Upstate Golisano Children’s Hospital, Upstate Medical University, Syracuse, NY 13210, USA; schaferm@upstate.edu (M.S.S.); ostena@upstate.edu (A.W.O.); 5 Department of Emergency Medicine, Norton College of Medicine, Upstate Golisano Children’s Hospital, Upstate Medical University, Syracuse, NY 13210, USA; 6 Department of Public Health and Preventive Medicine, Norton College of Medicine, Upstate Golisano Children’s Hospital, Upstate Medical University, Syracuse, NY 13210, USA

**Keywords:** appendicitis, post-operative, length of stay, laparoscopy, pediatrics, quality improvement

## Abstract

An uncomplicated appendectomy in children is common. Safely minimizing the post-operative length of stay is desirable from hospital, patient, and parent perspectives. In response to an overly long mean length of stay following uncomplicated appendectomies in children of 2.5 days, we developed clinical pathways with the goal of safely reducing this time to 2.0 or fewer days. The project was conducted in an urban, academic children’s hospital. The pathways emphasized the use of oral, non-narcotic pain medications; the education of parents and caregivers about expectations regarding pain control, oral food intake, and mobility; and the avoidance of routine post-operative antibiotic use. A convenience sample of 46 patients aged 3–16 years old was included to evaluate the safety and efficacy of the intervention. The mean post-operative length of stay was successfully reduced by 80% to 0.5 days without appreciable complications associated with earlier discharge. The hospital length of stay following an uncomplicated appendectomy in children may be successfully and safely reduced through the use of carefully devised, well-defined, well-disseminated clinical pathways.

## 1. Introduction

In the United States, an appendectomy for acute appendicitis is the most common surgical procedure among pediatric patients less than 17 years of age, with over 42,000 cases resulting in hospitalization in 2018 [1]. The nationwide incidence of appendicitis for both children and adults is estimated to be 108 per 100,000, with a 6% lifetime risk [2].

Hospitalization costs following an uncomplicated or complicated appendectomy vary from an estimated USD 6000 to USD 15,500, costing an estimated three billion annually [2,3]. The estimated cost of an uncomplicated appendectomy per encounter is variable in the literature. The median cost for an appendectomy in New York State is estimated to be USD 6170 [4]. Patients who required hospitalization were found to have higher total costs, including the additional costs of imaging, laboratory, and pharmacy services, compared to those not admitted [5,6].

The incidence of acute appendicitis in children has increased by 13.4% since 2020, with three in four cases classified as uncomplicated appendicitis [7]. An uncomplicated appendectomy is defined as the surgical removal of a non-ruptured appendix or the absence of a fecalith, abscess, visible hole, or purulent fluid in the abdomen [8]. In contrast, the surgical removal of an appendix that results in contamination of the peritoneal cavity or pelvis is defined as a complicated appendectomy [8]. While management for appendicitis can vary between operative management and antimicrobial therapy, in children, the standard of care typically results in the removal of the appendix laparoscopically due to a high failure rate and prolonged length of stay for nonoperative management. The Comparison of Outcomes of Antibiotic Drugs and Appendectomy (CODA) trial identified surgical intervention as the preferred treatment, as non-operative management resulted in an appendectomy nearly 30% of the time and increased both the prevalence of complications and the rate of serious adverse events [9]. Children who present with a higher initial pain score are 22% more likely to require an appendectomy [10]. Non-surgical treatment may be reserved for children who have comorbidities where the risk of anesthesia and surgery may outweigh the perceived benefits.

Previous studies have reported an average length of stay of 1.5 days for pediatric patients following an uncomplicated appendectomy; however, research is emerging that is putting into question the need for extended post-operative monitoring [11]. Even small but safe reductions in the post-operative length of stay per patient may be associated with significant reductions in hospital bed occupancy. Further, the hospitalization of pediatric patients places tension on the family system, including medical traumatic stress, parental anxiety and depression, financial strain due to loss of work and medical bills, academic impact due to loss of school time, and a risk of nosocomial infections [12,13]. Thus, a safe reduction in the post-operative length of stay, defined by the American College of Surgeons as the time between surgery and discharge, would benefit patients, families, and hospitals [8].

Broad consensus guidelines regarding multimodal perioperative care pathways, known as the Enhanced Recovery After Surgery program, have been developed and have shown improvements in the length of stay, reduced cost, and improved mortality [14,15,16]. Although the development and utilization of care pathways for children with uncomplicated appendicitis are likely areas of opportunity for the future, they were not available at the time of this study.

Our mean length of stay following an uncomplicated appendectomy in our children’s hospital was 2.5 days. Recent studies have demonstrated reductions in the length of stay following an uncomplicated appendectomy using clinical pathways [17,18,19]. We hypothesized that the creation of a post-operative clinical pathway process for children following an uncomplicated appendectomy at our institution would drive a reduction in the mean post-operative length of stay to 2.0 or fewer days. The primary objective of this initiative was to reduce the post-operative length of stay for pediatric patients following an uncomplicated appendectomy.

## 2. Materials and Methods

### 2.1. Context

We conducted this project at a large, 752-bed academic medical center located in an urban community in upstate New York that includes a 71-bed Children’s Hospital. This is the only tertiary care facility for pediatrics in the region and serves nineteen counties within the state. The institution achieved Magnet designation in 2021 and is verified as a Level 1 Pediatric Trauma Center through the American College of Surgeons and as a Pediatric Burn Center through the American Burn Association. Over 100,000 pediatric encounters are provided annually, with over 1200 requiring surgical care. Essentially all pediatric appendectomies in our region are performed by our pediatric surgery team, which is composed of five pediatric surgical attendings, three resident physicians in various years of training, and one pediatric nurse practitioner, working in the dedicated institutional Center for Children’s Surgery.

The primary goal of the Center for Children’s Surgery is to promote an environment in which the multidisciplinary perianesthesia team delivers quality care to a diverse pediatric population. The pediatric surgical services offered include pediatric general surgery, orthopedics, neurosurgery, urology, otolaryngology, trauma, burn, and gastrointestinal surgery. The site is open Monday–Friday from 06:45 to 19:30. An on-call pediatric team (pediatric surgeon, pediatric anesthesia, and pediatric nursing) is available outside of these hours. In addition, this team is on call from 18:30 on Friday evening until 07:00 on Monday morning. Staffing levels are based on the American College of Surgeons’ requirements and are adjusted as patient care needs change or case volumes change.

The institution annually reports surgical quality statistics and data to the American College of Surgeons through its National Surgical Quality Improvement Program (NSQIP) Pediatric [20]. According to our institution’s 2021 NSQIP Pediatric report, 194 appendectomies were performed in pediatric patients in that year; 137 of these were classified as uncomplicated [21]. Children who underwent an uncomplicated appendectomy at our institution in 2021 had zero surgical site infections, zero readmissions, and four post-discharge emergency department encounters. The mean length of post-operative hospital stay was 2.5 days. Our institution was therefore identified as a “high” outlier for length of stay following an uncomplicated appendectomy in comparison with the average length of stay for participating organizations in NSQIP Pediatric, which was reported as being between 0 and 2.0 days.

### 2.2. Study Goals

Given their successful use elsewhere, we endeavored to create clinical pathways to achieve reductions in the length of stay following an uncomplicated appendectomy based on identifying and addressing barriers to discharge. We used NSQIP Pediatric comparisons to arrive at the goal of reducing the length of stay from 2.5 days to 2.0 or fewer days. As balancing measures, we also examined the frequency of emergency department encounters or hospital readmissions within seven days of surgery.

Following pathway development and implementation, one of the authors (M.A.J.) reviewed and collected data from patients who underwent an uncomplicated appendectomy. We compared hospital-related patient charges before and after pathway implementation. Further, all patient families were contacted by a surgical data collection representative by telephone 24–48 h after hospital discharge and again about 30 days after hospital discharge.

### 2.3. Identification of Barriers to Discharge

**Figure 1:**
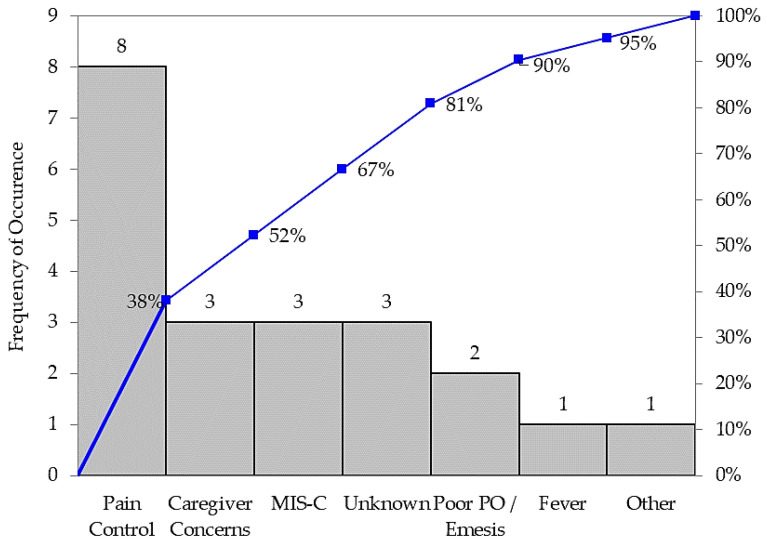
Previous barriers to discharge: Pareto chart.

A pediatric clinical nurse specialist (M.A.J.) and pediatric surgeon (J.D.S.), both with experience in quality improvement, reviewed each 2021 case of children with a prolonged length of stay following an uncomplicated appendectomy, enumerating a set of barriers to early discharge (Figure 1). Poor pain control, as identified by patient reports, emerged as the most common theme. Caregiver concerns, most often about eating solid food or patient activity, were the next most common. Additionally, caregivers’ perceptions of the expected length of stay following an appendectomy often contributed to hesitation around same-day discharge. Three of the patients in this cohort presented with a mixed clinical picture of appendicitis and multisystem inflammatory syndrome in children (MIS-C). These patients required additional hospitalization for up to seven days for MIS-C management, including steroids and intravenous immunoglobulin, following their appendectomy. Then, the COVID-related complication of multisystem inflammatory syndrome in children (MIS-C) was identified as the next most common barrier to discharge. Poor feeding and fever/infection concerns were also identified. Most patients were afebrile (68.7%) and did not require antibiotics (75%) post-operatively, tolerating oral fluids (87.5%) and ambulating independently (87.5%). Fever was defined as less than 38.5 degrees Celsius. Inconsistent responses to each of these concerns over the course of the year were apparent to the reviewers. Additionally, a lack of standardized post-operative management and discharge criteria highlighted the variability amongst the pediatric surgical team regarding the plan of care following an appendectomy.

### 2.4. Protocol Development

**Figure 2:**
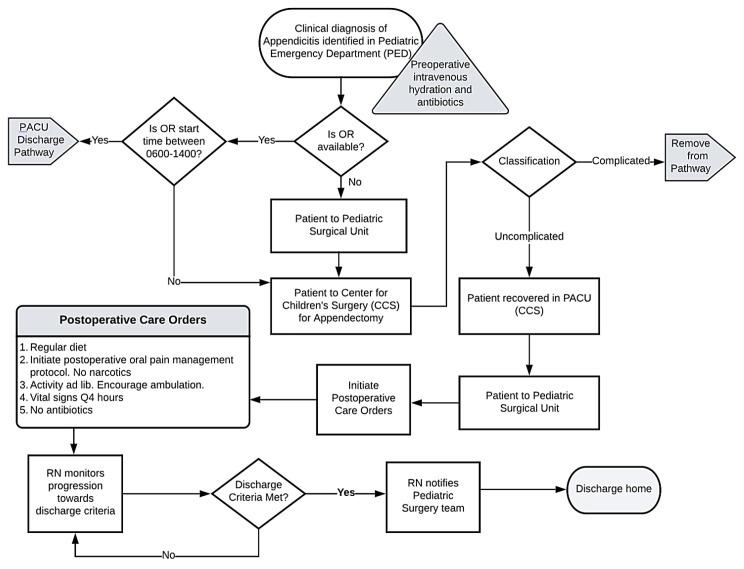
Clinical pathway flow diagrams for management and discharge of uncomplicated pediatric appendicitis from inpatient setting.

**Figure 3:**
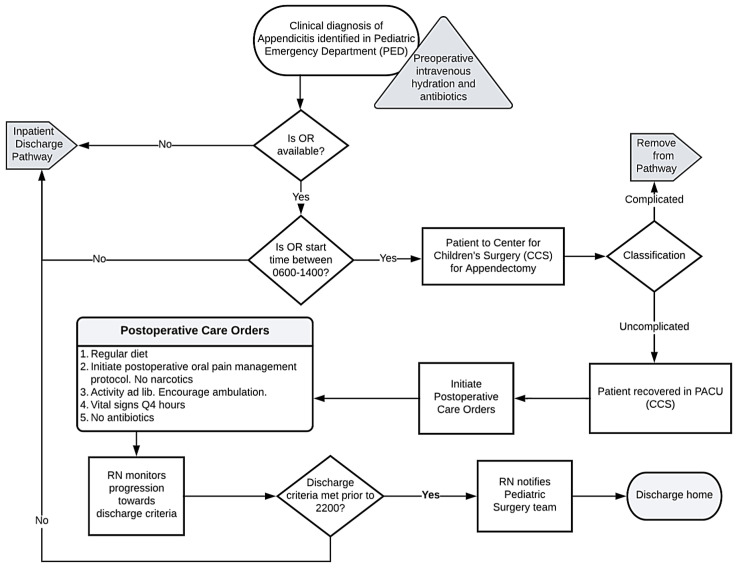
Clinical pathway flow diagrams for management and discharge of uncomplicated pediatric appendicitis from Post-Anesthesia Care Unit (PACU) setting.

A literature review was completed to discover standards of care and best practice recommendations. These results were reviewed with an interdisciplinary team, including our pediatric surgeons, pharmacy, and nursing leadership from the pediatric inpatient surgery, emergency department, and perioperative services. We then developed specific protocols to address the identified barriers by coming to a series of consensus decisions regarding the best practices to address each barrier to discharge. The developed protocol included an emphasis on oral, non-narcotic pain medications, specifically acetaminophen and ibuprofen. Acetaminophen (15 mg/kg/dose; maximum 650 mg/dose) and ibuprofen (10 mg/kg/dose; maximum 400 mg/dose) were ordered by oral route every six hours for a total of eight doses. Nursing staff and caregivers were instructed to alternate these medications every three hours around the clock for the first 48 h, then change as needed. Overnight doses were optional depending on patient comfort. This multimodal pain management strategy was found to be successful in children following an appendectomy, thus avoiding opioid-based medications [22]. The protocol included the education of parents and other caregivers about expectations regarding pain control, oral food intake, and mobility to purposely create reasonable expectations. Additionally, we hoped this strategy would result in an early partnership with caregivers so that they may anticipate a discharge within 24 h, possibly the same day as surgery. Although pre-operative antibiotics were specified, post-operative antibiotic use was to be avoided unless specific indications arose, such as a gangrenous-appearing appendix. Discharge was to be considered once the child achieved adequate hydration orally, ambulated independently, had pain controlled by oral non-narcotic medications, voided without difficulty, and was afebrile, with caregiver discharge acceptance. The nursing care team was made aware of the discharge readiness criteria and was instructed to notify the pediatric surgical team once the goals were met. We created two similar care pathways, which differed only based on whether the patient was discharged directly from the Post-Anesthesia Care Unit (PACU) or was discharged following admission to an inpatient setting post-operatively. We developed flowsheets based on the pathways (Figure 2 and Figure 3). Discharge from the PACU was indicated as being preferred when appropriate. We anticipated this discharge site as a potential barrier due to normal business hour limitations. Surgical staff were systematically educated about the pathways, and copies of the flowsheets were disseminated widely. Electronic order entry system order sets were prepared, designed for ease of order selection consistent with the clinical pathways. Preference for scheduled non-narcotic analgesics was indicated in the prebuilt physician order set, while antibiotics and narcotics were excluded from the order set. The clinical pathways were then simultaneously initiated across the Center for Children’s Surgery, inpatient pediatric surgical unit, and Pediatric Emergency Department. Following the initiation of the clinical pathways, we reinforced their concepts through weekly rounding and presentations of progress during perioperative and surgical quality leadership meetings. Additionally, cases were reviewed and discussed amongst the pediatric surgical team to ensure the safe delivery of care.

### 2.5. Patient Discharge Following Pathway Implementation

Following pathway development and implementation, one of the authors (M.A.J.) reviewed and collected data from patients who underwent an uncomplicated appendectomy; this included patient age, gender, primary language, past medical history, length of stay, presentation and operative date and time, discharge date and time, post-operative pain medications, average post-operative pain score for the first 24 h, and pain management plan following discharge. In addition, the discharge criteria from the clinical pathway, including avoidance of narcotics and antibiotics, presence of fever, ability to void, tolerance of oral hydration, and independent ambulation, were assessed. Patients were assessed by telephone by one of the authors (M.A.J.) for seven days following discharge for ambulatory or emergency department encounters related to dehydration, poor pain control, difficulty urinating, or fever/infection. Further, all patient families were contacted by a surgical data collection representative by telephone 24–48 h after hospital discharge and again about 30 days after hospital discharge. For the statistical analysis, categorical data were compared using the chi-square test, as appropriate. Significance was set a priori to a *p* value of <0.05.

## 3. Results

**Figure 4:**
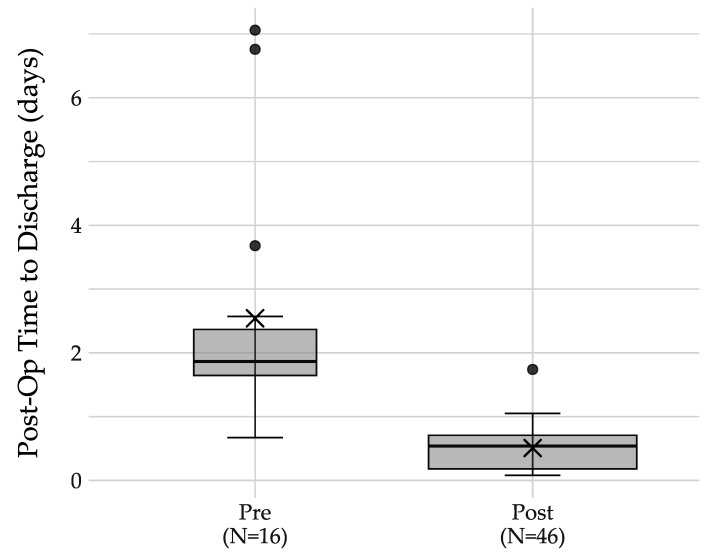
Distribution of post-operative length of stay: box plot.

A total of 61 appendectomies were performed in patients under 18 years of age. Fourteen patients were excluded due to a complicated appendectomy classification, and one patient was excluded due to an intraoperative complication. This resulted in 46 pediatric patients being eligible for the discharge clinical pathway. There were 46 uncomplicated appendectomies performed in patients under the age of 18 during the 90 days following the implementation of the clinical pathways. All of these patients were discharged in fewer than 2.0 days, with 95% being discharged in less than one day post-operatively. Four patients were discharged directly from the PACU. The restricted hours of the on-site PACU were a notable limitation in preventing hospital admission prior to discharge. We found a reduction in our mean post-operative length of stay from 2.5 to 0.5 days, which represents an 80% reduction (Figure 4). The range of the post-operative length of stay was 0.67 to 7.0 days in the pre-pathway cohort and reduced to 0.08 to 1.74 in the post-pathway cohort. The magnitude of the reduction in the post-operative length of stay was 2.0 days (95% CI 1.06–3.00, *p* = 0.0004). This exceeded the minimum project goal of a mean time of no more than 2.0 days.

We compared data from the 16 patients who underwent uncomplicated appendectomies followed by a prolonged hospital stay before the implementation of the pathways with those who underwent uncomplicated appendectomies following the implementation of the pathways. Of those 16 pre-pathway patients, 11 (68.7%) had appendectomies performed on weekdays, and 5 (31.2%) were performed on weekends. Of those children, 5 (31.2%) arrived at the PACU during day shift hours, and 11 (68.7%) arrived during evening shift hours. After the pathway implementation, 32 (69.5%) of the 46 patients who underwent uncomplicated appendectomies had their surgery performed on weekdays, and 14 (30.4%) were performed on weekends; 21 (45.6%) were performed during day shift hours, 07:00 to 18:00, while 25 (54.3%) were performed during evening hours. Three patients were considered outliers with a length of stay of up to seven days due to diagnostic uncertainty related to MIS-C. Demographic data regarding the uncomplicated appendectomy patients studied before and after the protocol implementation are included in Table 1.

Adherence to the discharge criteria from the clinical pathway was assessed for all 46 patients. All the patients remained afebrile post-operatively, sustained hydration orally, and voided without difficulty (Table 2). One patient experienced delayed ambulation due to poor pain control, requiring narcotics, while all the others were out of bed with minimal assistance and pain managed with acetaminophen and/or ibuprofen. Pain was adequately controlled, as identified as an average pain rating of less than or equal to three (on a pain scale from zero, “no pain”, to ten) in 78% of patients; no patients had a mean pain score greater than five. Ten patients were discharged in ≤4 h post-operatively. The patients who underwent an appendectomy on the weekend or after 1300 were more likely to be admitted and discharged the following morning, resulting in a post-operative length of stay of ≥12 h. Post-operative intravenous antimicrobials were ordered in two cases, both being children whose appendices were described as gangrenous. By definition, a gangrenous appendix is considered a subjective finding and is included as uncomplicated in our NSQIP Pediatric data [8]. While there is evidence that a gangrenous appendix may be safely managed as an uncomplicated appendicitis, the recommendation for or against antimicrobial use is inconclusive [23,24]. This assessment was therefore included as an uncomplicated appendicitis in our post-intervention sample. Following a discussion with our pediatric surgical team, we identified a gangrenous appendix as the only acceptable indication for post-operative antimicrobial use.

Balancing measures, collected to identify potential unintended adverse consequences of the implementation of the protocols, included Pediatric Emergency Department encounters and re-admissions within seven days of surgery. While patients were followed for a full 30 days following the surgical intervention through NSQIP Pediatric data, seven days were utilized for this initiative to assess the safety of the clinical pathway. Encounters due to poor pain control, dehydration, or difficulty voiding were anticipated to return within 7 days of discharge, rather than 30 days. Three patients presented to the Pediatric Emergency Department post-operatively; two of these patients required readmission. One of the readmission events was due to a pre-operatively existing influenza A infection. The second child was readmitted to the hospital for three days after presenting on post-operative day #2 with nausea and vomiting due to post-operative ileus. A third child presented post-operatively to the Pediatric Emergency Department after experiencing a syncopal episode; the child was deemed stable and was discharged home. While these encounters impacted the patient and family system, no patient harm was identified throughout the project.

## 4. Discussion

The goal of this project was to safely reduce the length of stay following an uncomplicated appendectomy in pediatric patients from 2.5 days to under 2.0 days through the creation and dissemination of two similar clinical pathways. These pathways emphasized the use of oral, non-narcotic pain medications, the avoidance of routinely ordered antibiotics, and the education of parents and caregivers about post-operative expectations. Their implementation was universally adopted and was associated with an 80% reduction in the post-operative length of stay to 0.5 days in 46 patients. This length of stay is consistent with national norms. Following hospital discharge, there were three Pediatric Emergency Department encounters, with two resulting in hospitalization, one of which was found to be surgically related. We are not aware of other adverse events. The population studied was demographically as expected for this condition and for the demographics of our local area.

Clinical pathways provide an opportunity to reduce variation in practice and standardize performance, thus improving patient outcomes [25]. The pathway implementation drove the surgical team to be consistent in their approach to pain, caregiver concerns, and other barriers. We anticipate improved sleep, behavior, and psychological impacts for the patients and their families. Families benefiting from a reduced length of post-operative hospital stay would also be expected to have reductions in the loss of school and work days, with reduced financial strain. A shorter average length of post-operative hospital stay also allows other patients and families to benefit from hospital services that might otherwise not be available, creating further positive downstream effects for the hospital system.

The discharge clinical pathway brought awareness to the need to standardize post-operative care for pediatric surgical patients. As the project progressed, the clinical pathway was published on the internal institutional website for easy access for staff. Staff reported the clinical pathway to be easy to follow and realistic to implement.

When considering the costs and benefits of a PACU discharge clinical pathway, the benefits outweigh the costs. This clinical pathway is built based on current staffing patterns and patient movement. Education materials were a minimal expense. The frequency of acetaminophen and ibuprofen use increased on the pathway, but the overall cost of post-operative medications decreased by avoiding antibiotics, IV analgesics, and antiemetics. Operative management alone is estimated to cost USD 3502 [26], while the overall hospital costs are estimated to decrease by not incurring an additional per bed per night charge of USD 7746 [27].

In addition to the fiscal advantage, a discharge within 24 h would likely result in increased patient and caregiver satisfaction as they could return to their home environment. By avoiding or reducing hospitalization, this will increase unit vacancy, which would allow for improved hospital throughput.

These results are concordant with the previously reported literature. In a 2019 review, it was reported that post-operative protocols and pathways with discharge criteria inclusive of oral pain management, oral hydration or regular diet, afebrile, antibiotic considerations, voiding, and ambulating independently successfully reduced the length of stay while enhancing overall satisfaction [28]. Gee et al. (2020) have reported successful post-operative pain management with acetaminophen and ibuprofen [22]. The same-day discharge of children after uncomplicated appendectomies has also been described [29]. Chisum et al. have reported a successful protocol to facilitate discharge for laparoscopic uncomplicated appendicitis; in contrast to our approach, however, their protocol was largely nurse-driven [19].

In addition to these appendicitis-focused studies, the implementation of care pathways to drive the high-quality delivery of perioperative care has been demonstrated for other common surgical conditions [30]. After the positive experience with the care pathway for an uncomplicated appendectomy, both staff and pediatric surgeons discussed the opportunity to develop additional pathways for other common surgical procedures.

It was unanticipated that only eight percent of the post-intervention sample would be discharged from the PACU. The surgical team would prefer that an uncomplicated appendectomy be an outpatient procedure, thereby avoiding inpatient hospitalization at all. However, since all the patients in the sample population presented through the Pediatric Emergency Department, ongoing efforts to reduce patient volumes in that area resulted in otherwise unnecessary pre-operative admissions. In addition, the patients often presented during weekdays after 1800, which often makes a same-day discharge unattainable due to normal business hours ending at 2200 for the Center for Children’s Surgery. Further work is needed on hospital flow to determine when a pre-operative admission is appropriate, when a patient should remain in the Pediatric Emergency Department until an operating room is available, and how to optimize operation timing to facilitate discharge from the PACU. The next steps for this care pathway include involving stakeholders from the Pediatric Emergency Department for a review of the data and an expansion of the care pathway to include pre-operative decision making.

### Limitations

The definition of “uncomplicated appendicitis” in this study was based on the visual appearance of the appendix, as documented in the operative note from the attending pediatric surgeon. For example, the appendix may appear to be uncomplicated during an intraoperative visual assessment, while micro-perforations may subsequently be identified in the surgical pathological specimen. In this way, the possibility of an inadvertent inclusion of a complicated appendix into our group of uncomplicated appendectomy patients cannot be excluded.

Our results reflect protocols developed and implemented at a single children’s hospital and may not be applicable to other institutions. Although ours is the only dedicated pediatric center in our region, the patients could potentially have sought post-operative care at other centers; however, no families described such events during the follow-up telephone calls. Further, our improvements in hospital discharge times were determined in comparison with past results; other environmental changes, such as factors associated with the COVID pandemic, might have also changed during that time, confounding our results. Specifically, the overlapping clinical presenting features and laboratory findings of MIS-C and appendicitis led to an increased length of stay for some 2021 patients, similar to cases reported in the literature [31,32,33]. While the inclusive population for the protocol was 0–17 years of age, this protocol was not assessed in patients under the age of 3 years due to the use of convenience sampling. Because of this, caution may be advised for children under this age. Finally, despite our attempts to identify adverse effects, there may potentially have been other unappreciated adverse effects of our protocols.

## 5. Conclusions

Using clinical pathways, we were able to safely reduce the length of stay following an uncomplicated appendectomy from 2.5 days to 0.5 days in pediatric patients. Future approaches will include continuing to refine and improve care pathways and expanding their use to other surgical conditions while monitoring for complications and reductions in patient/family/staff satisfaction.

## Figures and Tables

**Table 1:** Characteristics of uncomplicated appendectomy patients before and after implementation of care pathways.

Variable	Prior to Pathways (n = 16)	After Pathways (n = 46)	*p*-Value
Age			
≤10 years	6 (37.5%)	25 (54.3%)	0.25
11–14 years	7 (43.7%)	14 (30.4%)	0.36
≥15 years	3 (18.7%)	6 (13.0%)	0.60
Gender			
Male	11 (68.7%)	30 (65.2%)	0.80
Female	5 (31.2%)	16 (34.7%)	
Primary Language			
English	14 (87.5%)	43 (93.4%)	0.45
Other	2 (12.5%)	3 (6.5%)	
Previously Healthy			
Yes	15 (93.7%)	37 (80.4%)	0.21
No	1 (6.3%)	9 (19.5%)	

**Table 2:** Factors present impacting adherence to discharge pathway.

Present within 8 h Post Op	N = 46	%
Afebrile (<38.5)		
Yes	46	100
No	0	0
Intravenous Antibiotics		
Yes	2	4.3
No	44	95.6
Independent Ambulation		
Yes	45	97.8
No	1	2
Oral Hydration		
Yes	46	100
No	0	0
Voiding Without Difficulty		
Yes	46	100
No	0	0
Pain Management		
Non-narcotic agents	45	97.8
Narcotic agents	1	2

## Data Availability

Data is contained within the article.
